# Clonal Relatedness of Enterotoxigenic *Escherichia coli* (ETEC) Strains Expressing LT and CS17 Isolated from Children with Diarrhoea in La Paz, Bolivia

**DOI:** 10.1371/journal.pone.0018313

**Published:** 2011-11-29

**Authors:** Claudia Rodas, John D. Klena, Matilda Nicklasson, Volga Iniguez, Åsa Sjöling

**Affiliations:** 1 WHO Collaborating Centre for Research on Enterotoxigenic Escherichia coli (ETEC) and Department of Microbiology and Immunology, Institute of Biomedicine, University of Gothenburg, Gothenburg, Sweden; 2 U.S. Naval Medical Research Unit-3, Clinical Trials and Military Studies Program, Cairo, Egypt; 3 Instituto de Biología Molecular y Biotecnología, Universidad Mayor de San Andrés, Facultad de Ciencias Puras y Naturales, La Paz, Bolivia; University of Iowa, United States of America

## Abstract

**Background:**

Enterotoxigenic *Escherichia coli* (ETEC) is a major cause of traveller's and infantile diarrhoea in the developing world. ETEC produces two toxins, a heat-stable toxin (known as ST) and a heat-labile toxin (LT) and colonization factors that help the bacteria to attach to epithelial cells.

**Methodology/Principal Findings:**

In this study, we characterized a subset of ETEC clinical isolates recovered from Bolivian children under 5 years of age using a combination of multilocus sequence typing (MLST) analysis, virulence typing, serotyping and antimicrobial resistance test patterns in order to determine the genetic background of ETEC strains circulating in Bolivia. We found that strains expressing the heat-labile (LT) enterotoxin and colonization factor CS17 were common and belonged to several MLST sequence types but mainly to sequence type-423 and sequence type-443 (Achtman scheme). To further study the LT/CS17 strains we analysed the nucleotide sequence of the CS17 operon and compared the structure to LT/CS17 ETEC isolates from Bangladesh. Sequence analysis confirmed that all sequence type-423 strains from Bolivia had a single nucleotide polymorphism; SNP_bol_ in the CS17 operon that was also found in some other MLST sequence types from Bolivia but not in strains recovered from Bangladeshi children. The dominant ETEC clone in Bolivia (sequence type-423/SNP_bol_) was found to persist over multiple years and was associated with severe diarrhoea but these strains were variable with respect to antimicrobial resistance patterns.

**Conclusion/Significance:**

The results showed that although the LT/CS17 phenotype is common among ETEC strains in Bolivia, multiple clones, as determined by unique MLST sequence types, populate this phenotype. Our data also appear to suggest that acquisition and loss of antimicrobial resistance in LT-expressing CS17 ETEC clones is more dynamic than acquisition or loss of virulence factors.

## Introduction

One of the main pathogens that cause diarrhoea in humans is enterotoxigenic *Escherichia coli* (ETEC) [1]–[3]. Heat-labile toxin (LT) and heat-stable toxin (ST) are the main enterotoxins associated with ETEC-associated diarrhoea and phenotypic detection of one or both toxins or the genes encoding the toxins in isolates of *E. coli* is used to diagnose the infection [4], [5]. The ST toxin has been classified into two major genotypes, STh and STp [6], [7]. STh and STp are either expressed alone or in combination with LT, which may also be expressed alone [3], [5]. Human ETEC strains also produce one or more of several colonization factors (CFs) that mediate adherence to the small intestinal mucosa. Currently over 22 different CFs have been described for human ETEC including CFA/I, coli surface antigens (CS1) - CS8, CS12–CS15, and CS17–CS22 [5], [8]. The toxins and most CFs are known to be plasmid-borne [9]. ETEC strains are also classified by the conventional O∶H∶K serotyping scheme developed for *E. coli* where O serogroups are associated with cell wall lipopolysaccharides, H serogroups are antigenic determinants within flagella, and K antigens are capsular polysaccharide components [10]. Based on an extensive database analysis of ETEC from a number of different countries all over the world, Wolf reported that among the O, H, and K antigens, the O antigen component among ETEC isolates is most variable [11]. Enterotoxins, CFs, and O, H and K antigens are all exposed on the surface of ETEC, and therefore represent potential protective antigens and have been considered putative candidates for vaccine development against ETEC [11], [12].

To describe the genetic structure of microbial populations and the abundance of certain clones in a given environment, different molecular typing methods have been developed. Phylogenetic analyses play an important role in epidemiological studies since they may be used to address different kinds of questions such as if the isolates recovered from a localized outbreak of disease are the same or different strains (short term or local epidemiology) and how strains causing disease in one geographic area relate to those isolated world-wide (long term or global epidemiology). In both cases the methods used to describe the microbial population should be highly discriminatory since isolates assigned to the same molecular type are interpreted to have descended from a recent common ancestor, and isolates that fall into a different type share a more distant common ancestor. For bacterial pathogens, ribotyping, pulsed-field gel electrophoresis (PFGE), random amplified polymorphic DNA (RAPD) and multilocus sequencing typing (MLST) [13], [14] are commonly used to characterize populations. RAPD is a simple and fast method which can be applied to any species for which DNA can be extracted. Little knowledge of the underlying biochemistry or molecular biology of the species being studied is required. MLST is increasingly being applied to large collections of bacterial isolates for phylogenetic studies [15]. The method utilizes sequence data, primarily from housekeeping genes located in the chromosome, and affords direct interlaboratory comparisons since it does not depend on band positions and variances in electrophoretic conditions. Phylogenetic analyses using MLST and RAPD of ETEC strains have recently been used to determine the genetic variations and clonal lineages of this pathogen [13], [16]–[19]. Results have indicated that ETEC may have acquired virulence plasmids at several independent occasions during evolution and that the chromosomal genetic background is diverse and not specific for ETEC [17], [20].

In the present study we used MLST analysis to investigate the chromosomal genetic background of ETEC isolates obtained from a cohort study of Bolivian children aged less than 5 years with diarrhoea. During analysis of the toxin and CF profiles of ETEC isolates, it became apparent that the Bolivian ETEC collection presented an unusually high frequency of LT- and CS17-expressing isolates. Therefore, we determined if this was due to the emergence and circulation of a specific clone or if the LT/CS17 profile was found in multiple *E. coli* chromosomal genetic backgrounds, as determined by MLST, in Bolivia.

## Materials and Methods

### Ethical Statement

Ethical permission for the study was included as part of a Rotavirus Surveillance study in Bolivia obtained from the Comité Nacional de Bioéthica situated in La Paz, Bolivia. Parents seeking care for their children at several hospitals in La Paz and the closely located city El Alto that were enrolled in the study were asked upon arrival to the hospitals to give oral consent for analysis of stool samples from their children and for using anonymous clinical information. Ethical clearance for the Swedish part of the studies was given by The Regional Ethical Board of Gothenburg, Sweden (Ethics Committee Reference No: 088-10).

### Bacterial isolates and characteristics

Twenty-nine ETEC isolates recovered from children younger than 5 years old with watery, non-bloody diarrhoea were selected for MLST analysis from a cohort study performed from 2002 to early 2006 in La Paz, Bolivia [34]. In total the cohort study identified 79 children with ETEC diarrhoea from 853 stool samples. Of these, 11 isolates (14%) were assigned to be LT/CS17. These isolates were further analysed in this study together with 18 randomly selected ETEC isolates from the cohort study that expressed other toxin and colonisation factor profiles. In addition, four LT/CS17 isolates recovered from Bolivian children in the summer periods of 2007–2009 as a part of another study that identified 54 ETEC strains were analysed. Finally the CS17 encoding operon of 10 LT/CS17 ETEC isolates from Bangladeshi children younger than 5 years old recovered in 2003 as part of a large cohort study were analysed for comparison. All Bolivian isolates were initially analyzed for presence of ST and LT by GM1-ELISA and expression of CFs by dot blot assays. Results were further confirmed by both ELISA and multiplex PCR for expression of toxins and CFs as described previously [5], [21].

### Serogroup and antimicrobial resistance analysis

The 29 Bolivian isolates from 2002–2006 were analysed for serotype (O and H group) [22] using available O (O1–O185) and H (H1–H56) antisera in the *E. coli* Reference laboratory (LREC) at the University of Santiago de Compostela, Lugo, Spain. Isolates that did not react with any of the O and H antisera used were classified as nontypeable (ONT and HNT), and those that were nonmotile were denoted HNM.

Antimicrobial resistance to ten different antibiotics was tested as previously described [23], [24]. The 15 Bolivian LT/CS17 isolates from 2002–2009 were further analysed for 8 additional antibiotics (erythromycin, norfloxacine, penicillin G, oxacillin, cefadroxil, chloramphenicol, nitrofurantoin and trimethoprim) (Oxoid Hampshire; UK).

### Multilocus sequence typing (MLST) analysis

ETEC isolates were analyzed by MLST using sequenced internal regions of a set of seven housekeeping genes as described previously [25], [26]. Briefly, PCR products for the *E. coli* genes *adk* (adenylate kinase), *fumC* (fumarate hydratase), *gyrB* (DNA gyrase), *icd* (isocitrate/isopropylmalate dehydrogenase), *mdh* (malate dehydrogenase), *purA* (adenylosuccinate dehydrogenase), and *recA* (ATP/GTP-binding motif) were amplified for each isolate using the primers and PCR conditions described on the MLST website (http://mlst.ucc.ie/mlst). All PCR reactions were performed in 100 µl volumes using 10–100 ng of boiled bacterial DNA as the template. Negative (without template) controls were included in all experiments. PCR products were purified using the QIAquick® PCR Purification Kit (Qiagen, Hilden, Germany) and sequenced in both directions using the same primers as for PCR (MWG Biotech, Martinsried, Germany and GATC Biotech, Konstanz, Germany). DNA sequences from the ETEC isolates were assembled and aligned using ClustalX program within the Bioedit software package and subsequently the alleles from the ETEC isolates were edited to be the same length. Individual gene allele assignments were made by uploading the edited sequences into the *E. coli* MLST database. MLST sequence type assignments were made after interrogating the *E. coli* MLST database for combinations representing the seven assigned alleles. Dendrograms representing the relationship of the isolates from a concatenation of the MLST sequences from each isolate were constructed using the program Mega4 [27]. Phylogenetic reconstructions were created using the neighbour-joining method with the Kimura 2-parameter substitution model and branches were evaluated using the bootstrapping method with 1000 replications. Branch values below 70% were viewed as non-significant and are not shown in the dendrogram.

### Sequencing of the CS17 operon

PCR primers for amplification of regions within the *csb* operon, coding for the CS17 fimbrial gene cluster, were determined from the published sequence of ETEC strain WS6788A (GenBank no. AY515609) [28]. PCR was performed in a reaction mixture containing 1× PCR buffer, 1.5 mM MgCl_2_ and 5U *Taq* polymerase (Sigma-Aldrich CO, St. Louis, MO, USA), 0.2 mM dNTP (Roche Diagnostics Corp., Indianapolis, IN, USA), 0.4 µM of each primer (Table 1) and 5 µl DNA. The following programme was used: 94°C for 5 min, 30 cycles of 94°C for 30 s, 54°C for 30 s and 72°C for 1 min, followed by 72°C for 5 min. The PCR products of the major and minor subunits, the usher and the chaperone were initially sequenced in one direction using the respective forward primers (Table 1). The usher sequence was further amplified by the CS17-3874F and CS17-4517R primers and sequenced in both directions using these primers.

**Table 1:** PCR primers for amplification of the *csbB* (chaperone), *csbA* (major subunit), *csbC* (usher) and *csbD* (minor subunit).

Primer	Primer sequence 5′– 3′	Target
CS17-296F	gggcagttcaaaatggttgt	chaperone
CS17-2291R	ttctctccggaagcaagaaa	chaperone and major subunit
CS17-780F	cggtgcgtttaacacagcta	major subunit
CS17-3817F	gccggttacagttcgtcatt	minor subunit and 3′ end of usher
CS17-5729R	ataggcaggcgacaatcaac	minor subunit
CS17-1599F	ggggaagacgctgaatacaa	usher
CS17-4574R	acgaggggcttgaaaactct	usher

## Results

### ETEC in Bolivia represent known and novel *E. coli* MLST sequence types

**Figure 1:**
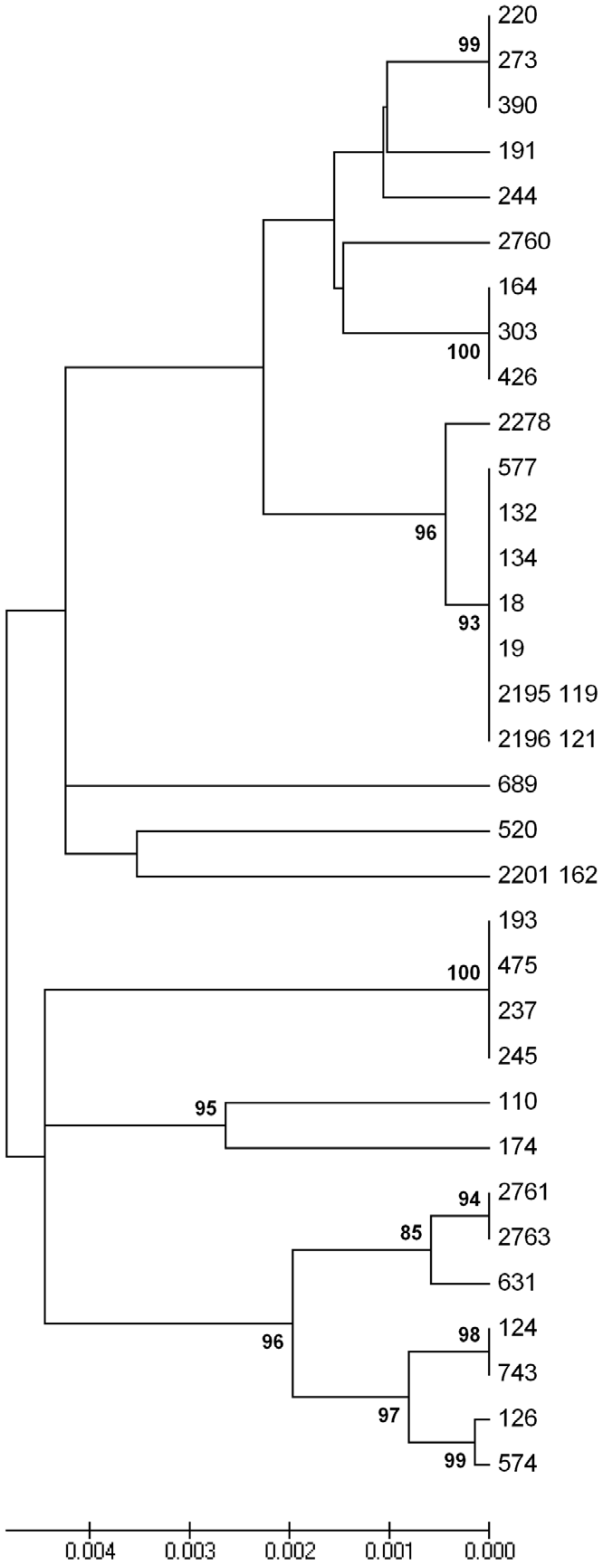
Dendogram of the 33 enterotoxigenic *Escherichia coli* strains from Bolivia included in the study.

Multilocus sequence typing of the 33 Bolivian ETEC isolates from 2002–2009 resulted in clustering of the samples into 18 MLST sequence types; 9 sequence types have been previously described by others (sequence type-423, -443, -173, -10, -165, -4, -648, -278 and -1139), and 9 allelic combinations resulting in new sequence types from Bolivia were recovered (Figure 1, Table 2, Table S1). The LT/CS17 isolates clustered into two major sequence types: sequence type-423 (n = 7), recovered between early 2002 to late 2005, and sequence type-443 (n = 3), isolated in 2003. Isolates expressing LT/CS17 recovered after 2005 (n = 5) represented four MLST sequence types; only one isolate (2278), recovered in 2007, was closely related to sequence type-423, differing at three nucleotide positions in the *gyrB* gene. The new LT/CS17 sequence type represented by isolate 2760 clustered close together with the sequence type-443 clone while isolates 2201 (162) and the two indistinguishable isolatess 2761 and 2763 had more unrelated sequence types (Fig. 1, Table 3). This showed that the Bolivian LT/CS17 strains belonged to several different MLST sequence types. The allele profiles of the LT/CS17 strains and the other Bolivian strains can be found in table S1.

**Table 2:** Characterised ETEC strains collected from Bolivian children with diarrhoea 2002–2009 organised with respect to genetic distance according to the MLST analysis and time of isolation within each sequence type.

Sequence type	Strain ID	Date of isolation	Toxin	CFs^a^	Serotype^b^	Antibiotic resistance^c^^, ^ d ^, ^ e
ST-173	220	16 May 02	LT	CS7	O114:H49	TSX
ST-173	390	14 Aug 02	LTSTh	CFA/I (CS21)	O78:HNM	-
ST-173	273	24 Sept 02	LT	-	O78: H-	nt
ST-1986	191	7 Jun 02	LT	-	ONT:H40	TET
ST-733	244	14 Aug 02	LTSTh	CFA/I CS21	O78:H-	AMP
ST-1988	2760	12 Mar 09	LT	CS17	Nd^f^	Nd^f^
ST-443	164	2 April 02	LT	CS17	Nd^f^	Nd^f^
ST-443	303	2 Jun 02	LT	CS17	Nd^f^	Nd^f^
ST-443	426	2 Aug 02	LT	CS17	Nd	Nd
ST-423	18	25 Feb 02	LT	CS17	O8:H9	AMP, CLOR, AMPS
ST-423	19	25 Feb 02	LT	CS17	O8:H9	AMP, CLOR, AMPS, TET
ST-423	577	5 March 03	LT	CS17	O8:H9	AMP, AMPS, TET, TSX
ST-423	119	15 Dec 05	LT	CS17	O6:H9	AMP, CLOR, AMPS
ST-423	121	16 Dec 05	LT	CS17	O8: H9	AMP, CLOR, AMPS
ST-423	134	21 Dec 05	LT	CS17	O8:H9	AMP, AMPS, TSX
ST-423	132	22 Dec 05	LT	CS17	O8:H9	AMP, AMPS, CLOR
ST-1990	2278	1 Nov 07	LT	CS17	nt	nt
ST-648	689	18 Jul 03	LTSTh	CS2 CS3	O1	AMP, AMPS
ST-1987	520	29 Dic 02	LT	-	ONT: HNM	AMP. AMPS, TET
ST-278	162	7 Jan 06	LT	CS17	O6: H16	AMP, AMPS
ST-731	193	7 Jun 02	LTSTh	CFA/I (CS21)	O78:HNM	AMP, AMPS, TSX
ST-731	245	14 Aug 02	LT	-	ONT:H40	-
ST-731	475	15 Oct 02	LT	CS1 CS3	ONT:H40	-
ST-731	237	5 Aug 02	LT	-	ONT:H40	-
ST-1989	110	Apr 02	LT	-	ONT:H-	nt
ST-1139	174	16 May 02	LTSTh	-	O8:H9	-
ST-1991	2761	1 Mar 09	LT	CS17	nt	nt
ST-1991	2763	14 Dec 08	LT	CS17	nt	nt
ST-4	631	28 April 03	LT	CS12	O159:H21	AMPS, TET, TSX
ST-10	124	19 Dec 05	LT	CS12	O159: H4	AMP, TET, TSX
ST-10	743	16 Sept 03	LT	CS12	O159:H4	-
ST-750	126	9 April 02	LT	-	O41:H32	-
ST-165	574	27 Feb 03	LT	-	ONT:HNM	-

a CS21 within brackets: positive for CS21 by PCR but negative in dot-blot.

b NT = Non typeable, HNM = nonmotile.

c Resistant to AMP = Ampicillin, AMPS = Amp-Sulbactam, CIP = Ciprofloxacin, CLOR = Chloramphenicol, GEN = Gentamicin, NAL = Nalidixic Acid, CEF = Cefoxitine.

TSX = Trimethoprim sulfamethoxazole, TET = Tetracyclin.

d nt = not tested.

e According to National Committee for Clinical Laboratory Standards for Antimicrobial Disk Susceptibility.

f Nd = not determined due to an initial co-infection of two strains in each patient; LT/CS17 and STh/CS6 strains were isolated, the LT CS17 strains persisted and were analysed in this study but we cannot rule out that the antibiotics profiling was performed on either or both strains at the time of testing.

**Table 3:** ST-423 strains and isolate 2278 organised with respect to time of isolation, antibiotic resistance pattern and clinical data.

ST type	StrainNo.	Age	Date of isolation	Sero group	Antibiotic resistance Tested 2010^a^	Clinical info
ST-423	18	15 m	25 Feb 02	O8:H9	ERY, PEN, OXA, CLO,	Outpatient without dehydration
ST-423	19	3 m	25 Feb 02	O8:H9	ERY, PEN, OXA, CLO	Outpatient with dehydration
ST-423	577	36 m	5 March 03	O8:H9	ERY, PEN, OXA, TM	Hospitalised with dehydration
ST-423	119	11 m	15 Dec 05	O6:H9	ERY, PEN, OXA, CLO	Hospitalised shock state
ST-423	121	11 m	16 Dec 05	O8:H9	ERY, PEN, OXA, CLO. TM	Hospitalised with dehydration
ST-423	134	24 m	21 Dec 05	O8:H9	ERY, PEN, OXA	Hospitalised with dehydration
ST-423	132	14 m	22 Dec 05	O8:H9	ERY, PEN, OXA, CLO	Hospitalised with dehydration
ST-1990	2278	10 m	1 Nov 07	nt	ERY, PEN, OXA, CLO	Hospitalised severe dehydration

a Resistance to Erythromycin (ERY), PenicillinG (PEN), Oxacilline (OXA), Chloramphenicol (CLO), Trimethoprim (TM), Norfloxacin, Cefadroxil and Nitrofurantoin was tested.

### Relationship of sequence type to serogroup, toxin and CF profile

Six of the seven LT/CS17 isolates belonging to the MLST sequence type-423 were serotype O8:H9; one isolate expressed O6:H9. The second major sequence type (sequence type-443) representing LT/CS17 isolates was not serotyped. Of the remaining five LT/CS17 isolates, three were non-typeable, one was O6:H16 and the serotype was not determined for the last isolate.

The second most abundant MLST sequence type was sequence type-731 (n = 4). Representative isolates expressed LT/STh or LT alone and either did not express or harbour any of the 19 CFs genes analysed, or phenotypically expressed CFA/I or CS1+CS3 (Fig. 1, Table 2). Only one isolate was O-typeable (O78) and only three were H-typeable (H40). Our data provides direct evidence that within a single MLST sequence type, differences in virulence profiles as well as serotypes can exist (Table 2).

### CS17 operon sequencing reveals a single nucleotide polymorphism in the usher sequence of the CS17 operon specific for a subset of Bolivian isolates

To further determine if the sequence type-423 LT/CS17 isolates could be genetically discriminated from the related 2278 isolate, we sequenced regions of the CS17 operon. Previously, we used this strategy to describe a large sequence variation in the operon of another CF, CS6 [19], [26]. We used the published sequence data for the genes of the CS17 operon *csbBACD* to obtain the sequence of the chaperone, major subunit, usher and minor subunit of the CS17 fimbria. Comparative analysis of the Bolivian CS17 isolates and Bangladeshi CS17 isolates revealed a single nucleotide polymorphism in the usher gene, *csbC*. The change, a single nucleotide polymorphism (SNP) of G-A, was located at position 4278 relative to W6788A and did not result in an amino acid change. The SNP was found only in sequence type-423 isolates and in two of the new MLST sequence types found in Bolivia. The CS17 isolates of other sequence types in Bolivia did not have this change, nor did any of the Bangladeshi isolates. We could find no record from any published sequence of a CS17 isolate or from ETEC expressing the genetically-related CFs CS19 and PCFO71 that had this specific nucleotide change. Hence the SNP was only found in the sequence type-423 Bolivian isolates and in isolates 2278, 2761 and 2763, we refer to this change as SNP_bol_.

### The antimicrobial resistance pattern within a clone is not stable

We now considered the LT/CS17 sequence type-423 O8:H9 isolates to be a clone based on identical MLST sequence types and identical virulence gene sequences. However, since strain 2278 shared the same CS17 operon sequence with this clonal group and only differed at one allele in the sequence type we suggest that this isolate recently evolved from the sequence type-423 clone. We examined the antibiotic resistance pattern of these strains as well as the other LT/CS17 strains to determine if the antimicrobial pattern was also conserved temporally in clones. Initial studies had confirmed a multi-resistance pattern in most of the ETEC isolates [23] (Table 2). We extended our original studies by analysing all 15 Bolivian LT/CS17 strains in parallel using an additional 8 new antimicrobial compounds. We also compared the time of isolation from patients and the reported clinical data regarding the diarrhoeal disease (Table 3). We found that the antimicrobial resistance pattern varied slightly between isolates which might indicate that clones gain or lose resistance genes more easily than they gain or lose the plasmids encoding the virulence factors. The clinical features of the children infected with the sequence type-423 clone and its single allele variant 2278 varied as well, indicating that host genetic factors might influence the disease outcome. Interestingly there was a trend of increasing severity of disease caused by the clone ranging from mild non-hospitalised in 2002 to severe diarrhoea in 2007 (Table 3).

## Discussion

Infection due to ETEC continues to be a major cause of childhood diarrhoea in developing countries, especially in children aged below 5 years. Studies world-wide often report that CFA/I and CS1-CS6 are the most common CFs; these surface antigens and the LT toxin are current targets for different vaccine strategies [11], [12], [29], [30]. The finding of an unexpectedly high number of LT/CS17 expressing ETEC isolates in Bolivia prompted us to further investigate whether this was due to a local clonal outbreak or to genetically unrelated isolates. We found by using MLST that at least two dominant MLST sequence types expressing LT and CS17 were present during several years in the La Paz area in Bolivia. However, several other sequence types were present among ETEC isolates expressing LT and CS17. These results indicate that several clones of LT/CS17 strains were circulating in Bolivia and that the high frequency of CS17 isolates was not due to a single clonal outbreak. In fact our results suggest that several sequence types expressing LT/CS17, such as isolates within the sequence type-423 and -443, co-circulate and that new sequence types emerge over time that share this phenotype. Indeed, ongoing studies in Bolivia seem to confirm that LT/CS17 isolates continue to commonly infect children and cause severe diarrhoea in Bolivia. This result is in contrast to findings in other parts of the world where LT-expressing isolates are considered in general to cause milder symptoms and less severe diarrhoea [29].

Several studies indicate that the chromosomal background, as measured by serogroup and RAPD, appears to correlate with and perhaps even determine toxin and CF profile in ETEC [11], [12], [18], [31]. This is in line with findings in the other pathogenic *E. coli* species, *e.g* EHEC and EPEC, where results indicate that these pathogens have evolved mainly by clonal expansion and that certain virulence factors, despite being plasmid borne, are only found in particular phylogenetic groups [20], [32]–[35]. However, the results of this study indicate that ETEC isolates with the same chromosomal background (*i.e.* MLST sequence type) may express different toxins and belong to different serogroups; we found that the same sequence type may be very variable with respect to toxin, CF and serogroup (Table 2). We have also found in this as well as in other studies that the same toxin/CF profile occurs in different MLST sequence types [26]. The diversity among ETEC is supported by recent studies in which ETEC toxins were found in all phylogenetic lineages of *E. coli* indicating that ETEC virulence genes may be acquired independently of the chromosomal background, although clonal expansion of particular strains occurs [20]. Hence whether an isolate is an ETEC seems to be defined entirely by the presence of toxins and to be less dependent on chromosomal background than is the case for other *E. coli* pathovars.

We define an ETEC clone as strains that share the same MLST sequence type, serogroup, toxin and CF profile but also identical sequences in their virulence genes. To convincingly determine whether the recovered LT/CS17 isolates from Bolivia were clonal we sequenced parts of the CS17 encoding operon in order to enhance genetic resolution. We found very little diversity in the CS17-fimbriae encoding operon sequence when comparing Bolivian and Bangladeshi isolates, although only a portion of the operon sequence was determined. One syntenic SNP in the coding region of the usher was uncovered. This SNP was detected in ten Bolivian strains but not in the five remaining Bolivian CS17 strains nor in any of the Bangladeshi strains. All strains belonging to sequence type-423, strain 2278, which had a sequence type related to 423, and two other LT/CS17 strains isolated in 2009 had the SNP. These results strongly supports that the sequence type-423 group contains strains that define a highly related genetic cluster. To this end we now consider the MLST sequence type-423 LT/CS17 O8:H9 SNP_Bol_ strains as a clone.

One might expect that clones evolve over time and in an attempt to follow if this was the case for the main Bolivian clone, we compared antibiotic resistance patterns as well as clinical symptoms of the children infected with the clone in order to propose a model of the circulation of this clone. When analysing phenotypic (serogroup and antimicrobial resistance patterns) and genetic traits (MLST and virulence gene SNPs) we found that clinical isolates 18 and 132 were indistinguishable. These ETEC were isolated almost four years apart, indicating that indistinguishable strains persist over time. Strains with sequence type-423 were all isolated between early 2002 and late 2005, and the related isolate 2278 was recovered in 2007 which may indicate that strains within this clonal group began drifting, at least in the *gyrB* gene, in 2006 or 2007. However at this stage we cannot rule out that isolate 2278 represents a new LT/CS17 clone. Isolates 2761 and 2763 were recovered in 2009 and had the Bolivian SNP but were phylogenetically distant from MLST sequence type-423, hence these strains might have acquired the Bolivian CS17 allele through horizontal transfer, perhaps from one of the older sequence type-423 clonal strains.

It is interesting to notice that the antimicrobial resistance patterns were stable with respect to resistance towards ampicillin, ampicillin–sulbactam, erythromycin, penicillinG and oxacillin but isolates seemed to gain or lose resistance genes towards chloramphenicol, tetracycline and trimethoprim more readily. Most isolates were multi-resistant, although the targets of these resistances were variable. This result further enforces the need for restricted use of antibiotics. In conclusion, we found that several LT/CS17 strains persist in Bolivia but that one clone, sequence type-423 O8: H9 SNP_Bol_, was repeatedly isolated from children. Another study has suggested that pathogenic *E. coli* clones may be increasingly resistant towards antibiotics and increasingly virulent over time [36]. Although further studies are needed to see if similar mechanisms are found in Bolivian ETEC clones we could see a tendency of increased virulence over time in MLST sequence type-423 O8: H9 SNP_Bol_ (Table 3). In conclusion this study is to our knowledge the first study to indicate that several different LT/CS17 clones circulate in Bolivia but that some individual clones such as MLST sequence type-423 O8: H9 SNP_Bol_ are more persistent than others and repeatedly infect children.

## Supporting Information

Table S1 Multi locus Sequence typing allele numbers of the strains included in the study. The alleles of the seven genes included in the Achtman *E. coli* scheme that designate the sequence type. New sequence types discovered were reported to the database and assigned new sequence type numbers. Clonal complexes includes related sequence types that differ from their nearest neighbour by no more than two of the seven loci.
(RTF)
