# Supplementary material for: Clonal Relatedness of Enterotoxigenic Escherichia coli (ETEC) Strains Expressing LT and CS17 Isolated from Children with Diarrhoea in La Paz, Bolivia
Source: PLoS One. 2011 Nov 29;6(11):e18313. doi: 10.1371/journal.pone.0018313 (PMC3226562; doi:10.1371/journal.pone.0018313)
Supplement: Table S1 — Multi locus Sequence typing allele numbers of the strains included in the study. The alleles of the seven genes included in the Achtman E. coli scheme that designate the sequence type. New sequence types discovered were reported to the database and assigned new sequence type numbers. Clonal complexes includes related sequence types that differ from their nearest neighbour by no more than two of the seven loci. (RTF) [file pone.0018313.s001.rtf]

Table S1.


Strain ID	adk	fumC	gyrB	icd	mdh	purA	recA	ST	Clonal complex	
220	6	6	15	16	42	46	7	ST-173	None	
390	6	6	15	16	42	46	7	ST-173	None	
273	6	6	15	16	42	46	7	ST-173	None	
191	6	6	15	16	17	46	7	ST-1986	None	
244	6	6	15	16	8	12	7	ST-733	None	
2760	243	6	5	16	11	8	7	ST-1988	None	
164	6	19	15	16	9	8	7	ST-443	ST205 complex	
303	6	19	15	16	9	8	7	ST-443	ST205 complex	
426	6	19	15	16	9	8	7	ST-443	ST205 complex	
18	6	4	33	1	20	12	7	ST-423	ST23 complex	
19	6	4	33	1	20	12	7	ST-423	ST23 complex	
577	6	4	33	1	20	12	7	ST-423	ST23 complex	
119	6	4	33	1	20	12	7	ST-423	ST23 complex	
121	6	4	33	1	20	12	7	ST-423	ST23 complex	
134	6	4	33	1	20	12	7	ST-423	ST23 complex	
132	6	4	33	1	20	12	7	ST-423	ST23 complex	
2278	6	4	5	1	20	12	7	ST-1990	None	
689	92	4	87	96	70	58	2	ST-648	None	
520	6	6	15	56	8	26	6	ST-1987	None	
162	9	23	64	18	11	8	6	ST-278	None	
193	6	122	57	1	8	8	81	ST731	None	
245	6	122	57	1	8	8	81	ST731	None	
475	6	122	57	1	8	8	81	ST731	None	
237	6	122	57	1	8	8	81	ST731	None	
110	10	7	1	8	8	8	6	ST-1989	None	
174	8	7	1	8	8	18	6	ST-1139	None	
2761	6	11	4	16	8	78	2	ST-1991	None	
2763	6	11	4	16	8	78	2	ST-1991	None	
631	6	5	4	8	8	8	2	ST-4	ST10 complex	
124	10	11	4	8	8	8	2	ST-10	ST-10 Complex	
743	10	11	4	8	8	8	2	ST-10	ST10 complex	
126	10	27	5	10	12	109	2	ST-750	None 	
574	10	27	5	10	12	8	2	ST-165	ST-165 Complex	
